# Identifying the Prognosis Factors and Predicting the Survival Probability in Patients with Non‐Metastatic Chondrosarcoma from the SEER Database

**DOI:** 10.1111/os.12521

**Published:** 2019-10-29

**Authors:** Runzhi Huang, Zhao Sun, Huimin Zheng, Penghui Yan, Peng Hu, Huabin Yin, Jie Zhang, Tong Meng, Zongqaing Huang

**Affiliations:** ^1^ Department of Orthopaedics The First Affiliated Hospital of Zhengzhou University Zhengzhou China; ^2^ Division of Spine Surgery, Department of Orthopaedics, Tongji Hospital Tongji University School of Medicine Shanghai China; ^3^ Key Laboratory of Spine and Spinal Cord Injury Repair and Regeneration, Tongji University Ministry of Education Shanghai China; ^4^ Department of Orthopedics, Shanghai Bone Tumor Institute, Shanghai General Hospital, School of Medicine Shanghai Jiaotong University Shanghai China; ^5^ Shanghai East Hospital, Key Laboratory of Arrhythmias, Ministry of Education Tongji University School of Medicine Shanghai China

**Keywords:** Bone cancer, Chondrosarcoma, Non‐metastatic, Prognostic factor, Survival analysis

## Abstract

**Objective:**

To identify prognostic factors and establish nomograms for predicting overall survival (OS) and cause specific survival (CSS) of patients with non‐metastatic chondrosarcoma.

**Methods:**

We collected information on patients with non‐metastatic chondrosarcoma from the Surveillance, Epidemiology, and End Results (SEER) database between 2005 and 2014, together with data from the First Affiliated Hospital of Zhengzhou University from 2011 to 2016. Variables including patients’ baseline demographics (age, race, and gender), tumor characteristics (tumor size and extension, histology subtype, primary site, and American Joint Committee on Cancer [AJCC] stage), therapy (surgery, chemotherapy, and radiotherapy), and socioeconomic status (SES) were extracted for further analysis. OS and CSS were retrieved as our researching endpoints. Patients from the database were regarded as the training set, and univariate analysis, Lasso regression and multivariate analysis as well as the random forest were used to explore the predictors and establish nomograms. To validate nomograms internally and externally, we applied bootstrapped validation internally with the training dataset, while the dataset for external validation was obtained from the First Affiliated Hospital of Zhengzhou University. We estimated the discriminative ability of nomograms based on Cox proportional hazard regression models by means of calibration curves and the concordance index (C‐index) of internal and external validation.

**Results:**

After the implementation of exclusion criteria, there were 1267 patients in the training set and 72 patients in the testing set with non‐metastatic chondrosarcomas. Age, gender, grade, histological subtype, primary site, surgery, radiation, chemotherapy, being employed/unemployed, tumor size, and tumor extension were significantly associated with prognosis in the univariate analysis. Age, gender, tumor size and extension, primary site, surgery, radiotherapy, chemotherapy, histological grade, and subtype were independent prognostic factors in the Cox models. The C‐index of nomograms (internal: OS, 0.787; CSS, 0.821; external: OS, 0.777; CSS, 0.821) were higher than following conventional systems: AJCC sixth (OS, 0.640; CSS, 0.673) and seventh edition (OS, 0.675; CSS, 0.711).

**Conclusions:**

Age, gender, tumor size and extension, surgery, histological grade, and subtype were independent prognostic factors for both OS and CSS. In addition, we revealed that chondrosarcomas in the trunk, radiotherapy, and chemotherapy were correlated with poor prognosis. Our nomograms based on significant clinicopathologic features can well predict the 3‐year and 5‐year survival probability of patients with non‐metastatic chondrosarcoma and assist oncologists in making accurate survival evaluation.

## Introduction

Chondrosarcoma, characterized by its ability to form cartilage, is the second most common primary malignancy of bone^1^, ^2^, ^3^. It often occurs in patients between 30 and 70 years of age, with the most frequent sites being the pelvis, the femur, and the shoulder girdle. In addition, it is generally slow‐growing and exhibits strong local aggressiveness. The aim of our multi‐institutional study is to identify prognostic factors and to establish nomograms for predicting overall survival (OS) and cause‐specific survival (CSS) of patients with non‐metastatic chondrosarcoma.

Surgical resection is the main treatment paradigm. However, anatomic constraints often hinder surgical outcomes and result in local recurrence. The therapeutic effects of radiotherapy and chemotherapy remain controversial^4^, ^5^, ^6^, ^7^. Clinical data shows that patients with locally advanced, unresectable or metastatic chondrosarcoma have poor survival outcomes. Thus, pre‐metastatic status is meaningful for early diagnosis and evaluating this status is helpful for improving prognosis. However, the evaluation of non‐metastatic tumors is still comparatively neglected in clinical practice. All the abovementioned problems lead to the unoptimistic situation of chondrosarcoma.

Identifying the prognostic factors of pre‐metastatic chondrosarcoma may help oncologists treat patients individually^8^, ^9^. To improve the predictive capability of survival models, previous studies constructed several nomograms, but most nomograms were derived from small samples in different research centers, which might diminish the accuracy of the predictors. Moreover, it is critical to apply external validation to evaluate generalizability and avoid overfitting, but none of them tried to validate externally with multi‐institutional databases, which decreases the credibility of the predication models^10^, ^11^.

The Surveillance, Epidemiology, and End Results (SEER) database currently collects and publishes data on cancer incidence and survival from 17 population‐based cancer registries encompassing approximately 26% of the US population. These specific local registries were chosen for their completeness and their adequate representation of minority populations^2^, ^3^, ^13^.

Metastasis in advanced stage cancer suggests a poor prognosis; therefore, early diagnosis in the pre‐metastatic stage is significant and evaluating this stage is helpful for improving prognoses^8^, ^12^, ^13^. However, a multi‐center study based on a large sample size exploring the prognostic factors of non‐metastatic chondrosarcomas has not been reported. In this study, we first selected patients with non‐metastatic chondrosarcoma from the SEER database. Machine learning and classic regression analysis were used to identify independent prognostic variables and nomograms were constructed to estimate the OS and CSS. American Joint Committee on Cancer (AJCC) sixth and seventh editions were compared with nomograms for predicting survival. Moreover, a high‐quality external validation was included in our study to evaluate the accuracy and applicability of the nomograms in clinical work.

## Material and Methods

### *Patients Selection*

On 15 July 2018, we selected patients diagnosed with chondrosarcoma histologically from the SEER database from 2005 to 2014. Patients who were not diagnosed with chondrosarcoma by biopsy, together with those who were not diagnosed with their first tumors and were not diagnosed with tumors at M0 or N0 stage, were excluded from our study. Patients with missing data (unknown tumor size/tumor extension/grade/race/marital status/surgery information) were also excluded.

### *Data Extraction*

The variables were obtained from the SEER database, including patients’ baseline demographics (age, race, and gender), tumor characteristics (tumor size and extension, histology subtype, primary site, and AJCC stage), therapy (surgery, chemotherapy, and radiotherapy), and socioeconomic status (SES). OS and CSS were retrieved as our researching endpoints. On the basis of the same eligibility criteria, we also extracted information on patients from the First Affiliated Hospital of Zhengzhou University between 2011 and 2016.

### *Definition, Measurement Method, and Clinical Significance of Each Variable*

The baseline demographics included age of diagnosis in years (representing the age of the patient at diagnosis for this cancer), race (white, black, and other), and gender.

The tumor size and tumor extension in millimeters represented the maximum diameter of the tumor and the tumor invasion region, respectively.

Histopathologically, patients were subdivided into seven subtypes on the basis of the ICD‐0‐3 coding system. Because there was only 1 patient with malignant chondroblastoma (9230/3) and there were only 8 patients with clear cell chondrosarcoma (9242/3), and they had similar outcomes of survival analysis to juxtacortical chondrosarcoma (9221/3), the cohort was categorized into five groups: no other specific (NOS) (9220/3), myxoid (9231/3), mesenchymal (9240/3), dedifferentiated (9243/3), and others (consisted of chondroblastoma, clear cell chondrosarcoma, and juxtacortical chondrosarcoma)^14^, ^15^.

The primary site of the tumor was defined as the site where the primary tumor occurred, and was divided into five levels: head, face, neck; lower limb; upper limb; thorax, abdomen; and trunk.

The AJCC staging system is the most commonly used tumor staging system around the world^14^. Data of the AJCC sixth edition is available from 2004 and the seventh edition is available from 2010 in the SEER database. The AJCC sixth edition data were used in the statistical analysis of the training and the testing set, while the AJCC seventh edition data were only applied to the comparison with our nomograms.

In terms of therapy, we extracted all information on treatment in the SEER database, including only whether or not to receive surgery (Surgery not performed and Surgery performed), radiotherapy (No/Unknown and Yes) and chemotherapy (No/Unknown and Yes)^15^.

Socioeconomic status is a composite measure of an individual's sociological and economic standing. In this study, SES included ninth grade education, high school education, at least bachelor degree, median family income, families below poverty, unemployment, and white collar. These seven SES variables incorporated into further analysis represented the percentage of the persons who were under one of these SES, calculated from the Census 2000–2015 American Community Survey (ACS) data in the SEER database. All the continuous SES variables were split into categorical variables by the medians.

### *Statistical Analysis*

As a descriptive statistic, we reported dichotomous variables as percentages while continuous variables as mean and median (range). Continuous variables were divided into categorical variables based on the mean multidimensional scaling (MDS) plot of the random forest (age: <65 years, ≥65 years; tumor size: <100 mm, ≥100 mm; tumor extension: <300 mm, ≥300 mm). Subsequently, we performed three statistical methods (parameter or non‐parametric test, Kaplan–Meier method, random forest [Ntree = 500]) to seek the most significant predictors associated with survival. In the random forest algorithm, variables’ contributions to the classification of the endpoints were ranked according to mean decrease Gini (MDG). Greater MDG suggested greater classification contribution. Out of bag (OOB) error rate was applied to evaluate the random forest's classification accuracy in our study.

Potential significant predictors were integrated into the Cox proportional hazards model. Lasso regression was performed to ensure that the multifactor models were not overfitting. We built the final models using only significant predictors of the initial multivariable models. Eventually, nomograms based on the final models were developed to predict OS and CSS. Calibration curves and C‐index of internal and external validation were used to evaluate the calibration of predictors and the discrimination ability of the models, respectively. In addition, to compare the predictive ability of the AJCC staging systems and the nomograms, we applied the Kaplan–Meier method and calculated the C‐index of AJCC sixth and seventh editions.

Only two‐sided *P*‐values less than 0.05 were considered statistically significant. All statistical analysis was conducted using R version 3.5.1 software (Institute for Statistics and Mathematics, Vienna, Austria; www.r-project.org).

### *Ethical Approval and Informed Consent*

The study was approved by the Ethics Committee of the First Affiliated Hospital of Zhengzhou University (No. KEYAN‐2018‐LW‐021). Informed consent was obtained from all individual participants included in the study.

## Results

### *Patient Characteristics*

**Figure 1:**
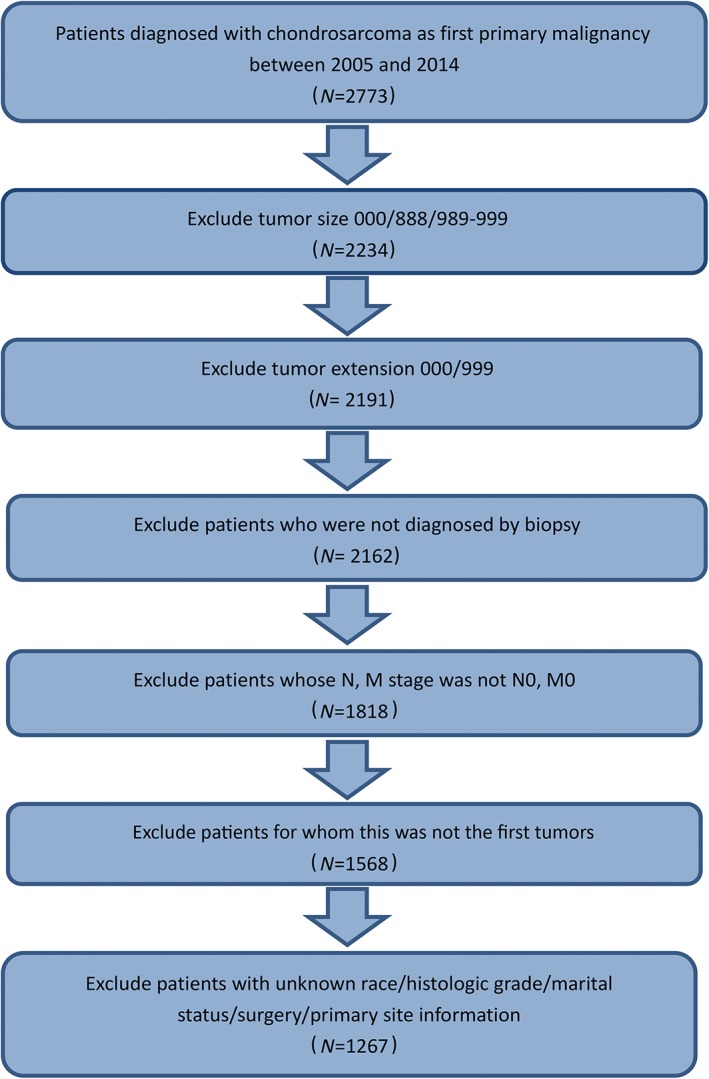
Flow chart of patient selection from the Surveillance, Epidemiology, and End Results (SEER) database.

A total of 2773 patients with chondrosarcoma were selected from the SEER database and 1267 patients were left in our cohort after elimination. Figure 1 shows the process of data selection. Patient characteristics are described in Table S1. Of the total 1267 patients, age, gender, grade, histological subtype, primary site, surgery, radiation, chemotherapy, being employed/unemployed, tumor size, and tumor extension were significantly different between the alive cohort and the dead cohort. Among the patients, 721 (56.9%) were male and 546 (43.1%) were female. The population with a median age of 51.00 (range, 4.00–91.00) years and White people (87.1%) predominated. These non‐metastatic chondrosarcomas were dominantly T1 (AJCC T stage) (60.9%), grade II (42.8%), and NOS histologically (78.2%), with the median size of 65.00 (range, 4.00–890.00) mm and the median extension of 300.00 (range, 100.00–850.00) mm. The median survival was 42.00 (range, 0.00–119.00) months. At the endpoints, 149 (9.2%) and 202 (15.9%) patients died of specific and all causes, respectively.

Table S15 summarizes the results of descriptive statistics for the validation set. The results of the nonparametric test also showed demographic homogeneity between the validation set and the training set (Categorical age: *P* = 0.155; Gender: *P* = 0.250). However, statistically significant heterogeneity was found in tumor extension (*P* < 0.001), primary site (*P* < 0.001), grade (*P* < 0.001), ICD‐O‐3 histology subtype (*P* < 0.001), surgery information (*P* < 0.001), chemotherapy (*P* < 0.001), and follow‐up time (*P* < 0.001) between the two sets.

### *Univariate Analysis and Random Forest*

Results of random forest and univariate analysis are shown in Table S2. Both Kaplan–Meier analysis and parametric or non‐parameter tests indicated that age, gender, grade, histological subtype, primary site, surgery, radiation, chemotherapy, being employed/unemployed, tumor size, and tumor extension were associated with patient prognosis. In addition, these variables also had relatively high MDG in the random forest. Hence, these variables were included in the initial multivariable models.

### *Cox Proportional Hazards Model and Lasso Regression*

**Figure 2:**
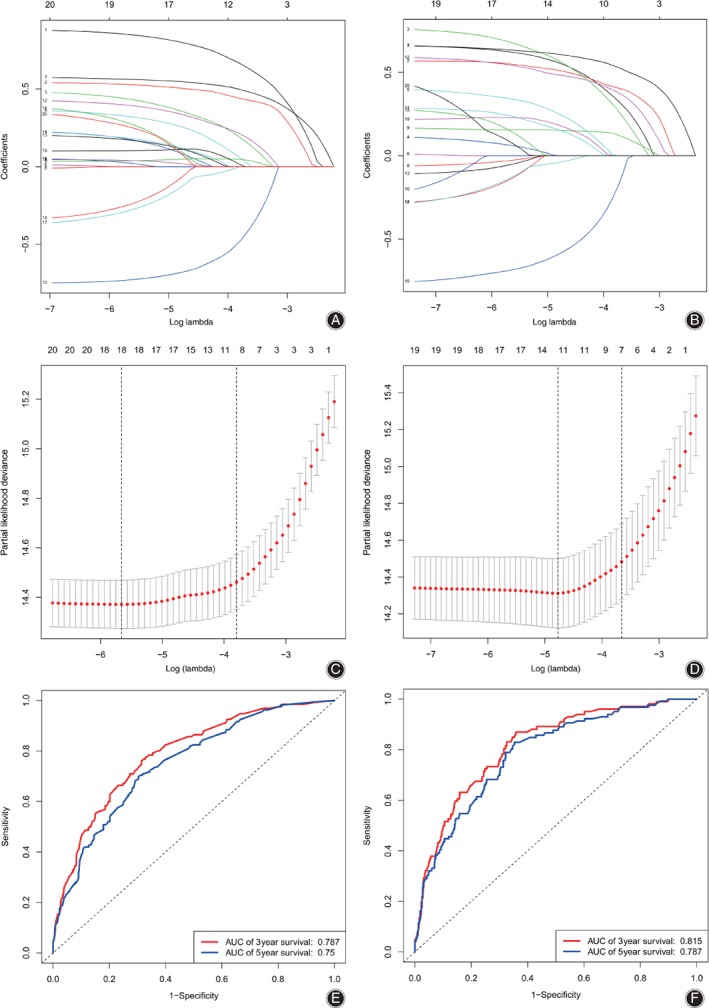
The results of the lasso regression (A–D) and the receiver operating characteristic curves (E, F).

The results of final Cox proportional hazard regression models, only consisting of significant predictors of the initial multivariable models, are presented in Table 1. To avoid overfitting, lasso regression suggested including 8 and 11 variables when overall survival (OS) and cause‐specific survival (CSS) was the endpoint, respectively (Fig. 2a, b, c, d). Referring to patients <65 years old, the senior age group had poorer OS (≥65 years: HR, 2.121; 95% CI, 1.586 to 2.836; *P* < 0.001) and CSS (1.886; 1.323 to 2.689; *P* < 0.001). Patients with larger tumor size and extension had worse prognosis according to OS (size ≥100 mm: 1.782; 1.333 to 2.381; *P* < 0.001) (extension ≥300 mm: 1.688; 1.156 to 2.465; *P* < 0.001) and CSS (size ≥100 mm: 1.725; 1.207 to 2.467; *P* < 0.001) (extension ≥300 mm: 2.394; 1.451 to 3.948; *P* < 0.001). Compared with females, males had worse OS (1.609; 1.195 to 2.165; *P* = 0.002) and CSS (1.552; 1.084 to 2.221; *P* = 0.016). Moreover, relative high grade was associated with poor survival (OS: grade III: 2.764; 1.742 to 4.386; *P* < 0.001; grade IV, 3.854; 2.259 to 6.574; *P* < 0.001) (CSS: grade III: 2.879; 1.653 to 5.013; *P* < 0.001; grade IV: 4.024; 2.148 to 7.540; *P* < 0.001). Only dedifferentiated chondrosarcoma was significant in histology subtypes, indicating worse OS (2.737; 1.748 to 4.285; *P* < 0.001) and CSS (2.548; 1.536 to 4.228; *P* < 0.001). Surgery was an independently protective factor (OS: 0.365; 0.225 to 0.590; *P* < 0.001; CSS: 0.486; 0.267 to 0.884; *P* = 0.018), while radiation was a risk factor of CSS (1.507; 1.005 to 2.260; *P* = 0.047). Figure 2e and f shows the receiver operating characteristic curves (ROC) suggesting that the multivariate models had high accuracy (OS: area under curve [AUC] of 3‐year survival: 0.787; AUC of 5‐year survival: 0.750) (CSS: AUC of 3‐year survival: 0.815; AUC of 5‐year survival: 0.787).

**Table 1:** Cox proportional hazards regression model for overall survival and cause‐specific survival in patients with non‐metastatic chondrosarcoma

Variable	Overall survival (OS) Hazard ratio (95% CI)	*P* value	Cancer specific survival (CCS)	
			Hazard ratio (95% CI)	*P* value
Categorical age				
<65	1.000 (reference)		1.000 (reference)	
≥65	2.121 (1.586 to 2.836)	<0.001*	1.886 (1.323 to 2.689)	<0.001*
Categorical tumor size				
<100 mm	1.000 (reference)		1.000 (reference)	
≥100 mm	1.782 (1.333 to 2.381)	<0.001*	1.725 (1.207 to 2.467)	0.003*
Categorical tumor extension				
<300 mm	1.000 (reference)		1.000 (reference)	
≥300 mm	1.688 (1.156 to 2.465)	0.007*	2.394 (1.451 to 3.948)	0.001*
Gender				
Female	1.000 (reference)		1.000 (reference)	
Male	1.609 (1.195 to 2.165)	0.002*	1.552 (1.084 to 2.221)	0.016*
Primary site				
Head, face, neck			1.000 (reference)	
Lower limb			1.898 (0.820 to 4.395)	0.135
Thorax, abdomen			0.969 (0.404 to 2.328)	0.945
Trunk			2.098 (0.897 to 4.907)	0.087
Upper limb			1.070 (0.425 to 2.694)	0.887
Grade				
Grade I	1.000 (reference)		1.000 (reference)	
Grade II	1.486 (0.994 to 2.220)	0.053	1.362 (0.832 to 2.227)	0.219
Grade III	2.764 (1.742 to 4.386)	<0.001*	2.879 (1.653 to 5.013)	<0.001*
Grade IV	3.854 (2.259 to 6.574)	<0.001*	4.024 (2.148 to 7.540)	<0.001*
Histological subtype				
Chondrosarcoma, NOS	1.000 (reference)		1.000 (reference)	
Dedifferentiated chondrosarcoma	2.737 (1.748 to 4.285)	<0.001*	2.548 (1.536 to 4.228)	<0.001*
Mesenchymal chondrosarcoma	1.275 (0.600 to 2.710)	0.527	1.013 (0.405 to 2.531)	0.979
Myxoid chondrosarcoma	1.202 (0.794 to 1.820)	0.384	0.891 (0.528 to 1.505)	0.667
Others	0.465 (0.114 to 1.900)	0.286	0.270 (0.037 to 1.962)	0.195
Surgery information				
Surgery not performed	1.000 (reference)		1.000 (reference)	
Surgery performed	0.365 (0.225 to 0.590)	<0.001*	0.486 (0.267 to 0.884)	0.018*
Radiation				
No/Unknown			1.000 (reference)	
Yes			1.507 (1.005 to 2.260)	0.047*
Chemotherapy				
No/Unknown			1.000 (reference)	
Yes			1.508 (0.927 to 2.453)	0.098

CSS, cause‐specific survival; OS, overall survival

* *P* < 0.05.

In addition, as a sensitivity analysis, ANOVA was applied to compare the initial and final multivariable models, showing no significant results.

### *Nomogram and Validation*

**Figure 3:**
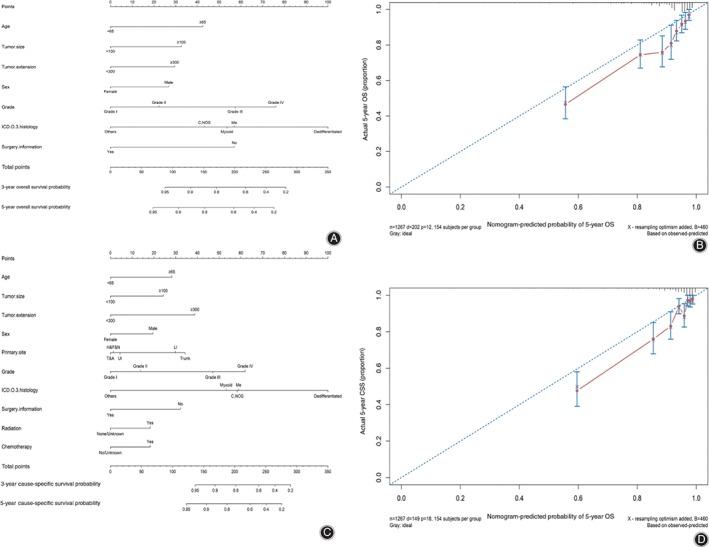
Nomograms and calibration curves predicting the probability of OS and CSS. (1) Nomograms for predicting 3‐year and 5‐year OS (A) and CSS (C) for patients with non‐metastatic chondrosarcoma. (2) Calibration curves showed the presentable accuracy of nomograms in predicting the 3‐and 5‐year OS (B) and CSS (D) by comparing nomogram predictions with actual endpoints. CSS, cause specific survival; H&F&N, head, face and neck; ICD, International Classification of Diseases; Ll, lower limb; Me, mesenchymal chondrosarcoma; C, NOS, chondrosarcoma, no other specific; OS, overall survival; T&A, thorax, abdomen; TNM, tumor node metastasis; Ul, upper limb.

**Figure 4:**
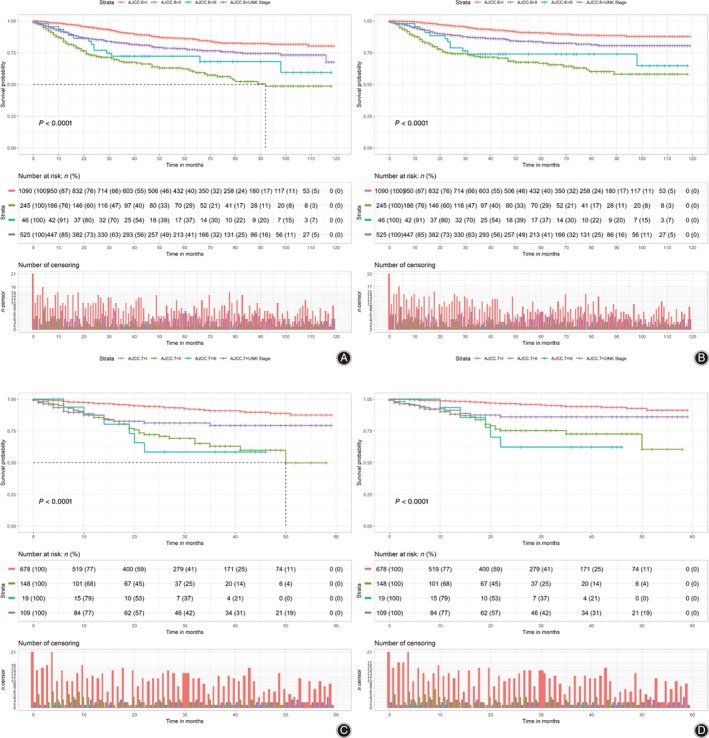
Kaplan–Meier curves of overall survival (OS) (left) and cause‐specific survival (CSS) (right) for the American Joint Committee on Cancer (AJCC) sixth and seventh edition staging system. (1) Kaplan–Meier analysis showed the correlation between the AJCC sixth edition staging system and the OS (*P* < 0.001, Fig. 4A) and CSS (*P* < 0.001, Fig. 4B) in patients with non‐metastatic chondrosarcoma. (2) Kaplan–Meier analysis showed the correlation between the AJCC seventh edition staging system and the OS (*P* < 0.001, Fig. 4C) and CSS (*P* < 0.001, Fig. 4D) in patients.

Nomograms based on final Cox models were constructed to evaluate predictive ability of the 3‐year and 5‐year OS and CSS with predictors in Fig. 3a and c. Table S3 shows the score of each predictor in nomograms. Then, to validate nomograms internally and externally, we applied bootstrapped validation internally with the training dataset, while the dataset for external validation was obtained from the First Affiliated Hospital of Zhengzhou University. The external validation set contained 72 patients (mean follow‐up time: 29.17 months; at least 2‐year follow up for each patient) and the median survival time was 20.50 (range, 1.00–92.00) months, along with the 90.3% and 88.9% for 3‐year and 5‐year survival probability, respectively. The C‐index for internal validation of OS and CSS was 0.787 and 0.821, respectively, while the C‐index for external validation of OS and CSS was 0.777 and 0.872, respectively. Meanwhile, we used the calibration plots (Fig. 3b and d) to validate the concordance of nomograms by comparing predicted values with the actual endpoints. To compare nomograms with traditional staging systems, we obtained the C‐index of AJCC sixth edition (OS, 0.675; CSS, 0.711) and the AJCC seventh edition (OS, 0.640; CSS, 0.673) (Fig. 4a‐d).

## Discussion

Chondrosarcoma is the second most frequent primary malignant tumor of the bone^1^, ^2^, ^16^. Metastatic chondrosarcoma is an advanced stage of this disease and has been demonstrated as a predictor of poor prognosis^8^, ^12^, ^13^. Thus, evaluating the pre‐metastatic stage is meaningful to improve prognosis and assist clinicians in making therapeutic choices. Comprehensive nomograms are useful and convenient tools to evaluate the prognosis of patients and they have been reported for some cancers^17^, ^18^, ^19^, ^20^. In this study, we first constructed prognostic nomograms for patients with non‐metastatic chondrosarcoma based on SEER database and validated them externally.

Our training cohort contained 721 (56.9%) males and 546 (43.1%) females, with a mean age of 50.50 (range, 4.00 to 91.00) years, similar to prior studies^11^, ^21^. In our series, we discovered that patients ≥65 years had poorer OS and CSS. Prior to our series, many studies drew the same conclusion^8^, ^11^, ^22^. The subgroup analysis implied that elder patients were prone to reject surgery (Table S4, *P* = 0.005), probably because they could not tolerate surgery well. Gender was also demonstrated to be a significant prognostic factor. We found that male patients tended to develop larger tumors (*P* < 0.001) and tumors were more likely to occur in the trunk (*P* = 0.003), where it was not easy to perform the en bloc tumor resection (Table S5–S6). Similar results were reported by Giuffrida *et al*.^13^ and Björnsson *et al*.^23^

In this study, we confirmed that tumor size ≥100 mm, tumor extension ≥300 mm, and increasing pathological grade suggested poor prognosis, which had been reported in many previous studies^13^, ^24^, ^25^. Larger tumor extension increased the difficulty and risk of surgical resection and, therefore, other adjuvant therapies should be performed to decrease tumor recurrence^9^. Moreover, larger tumor extension was reported to correlate with the overexpression of matrix metalloproteinases, which was meant to enhance tumor metastasis^26^. The in‐depth analysis revealed that patients with a larger infiltration range were more likely to reject surgery (Table S7, *P* = 0.045) and to have high histological grade (Table S8, *P* = 0.001).

In our study, 20.3% of chondrosarcomas occurred in the trunk (pelvis, spine, and scapula), suggesting poor prognosis. Previously, primary sites were often divided into two groups: axial and appendicular groups^11^, ^13^. Chondrosarcomas in the pelvis and spine had a worse prognosis due to anatomic constraints hindering the efforts of complete tumor resection, which resulted in a high rate of tumor recurrence^7^, ^8^, ^9^, ^24^, ^27^, ^28^. However, the subgroup analysis showed that surgery did not have an close connection with the primary site, because most of the patients underwent surgery no matter where tumors occurred (Table S9).

In the studies of chondrosarcoma, few studies have integrated all histology subtypes in statistical analysis. Although similar research by Song *et al*. analyzed this variable, it had limitations because patients with grade I and IV disease were excluded^11^. In this study, we confirmed that the dedifferentiated chondrosarcoma portended a worse outcome, which was in line with results of previous studies^4^, ^7^, ^11^.

Surgical resection is widely accepted as a significant protective factor for patients with chondrosarcoma, in line with our study^4^, ^9^, ^24^, ^29^. To our surprise, we found that chemotherapy was a significant risk factor both in univariate and multivariate analysis. In prior studies, chemotherapy exerted limited efficacy on most chondrosarcoma^5^, ^6^, ^30^, except for improving OS in mesenchymal chondrosarcoma^5^, ^31^. However, the therapeutic effect had not been confirmed yet, owing to lack of evidence from randomized controlled trials. Subsequently, we performed subgroup analysis and the results suggested that patients with chemotherapy were more likely to have higher grades (*P* < 0.001), greater tumor sizes (*P* < 0.001), and tumor extensions (*P* = 0.002) (Table S10–S12). Thus, we supposed that chemotherapy was not used in patients with non‐metastatic chondrosarcoma routinely and it was regarded as an adjuvant therapy for advanced chondrosarcoma, consistent with guidelines^1^.

The therapeutic effect of radiotherapy was controversial in previous studies. Krochak *et al*.^32^ and McNaney *et al*.^33^ reported that radiotherapy exerted limited efficacy, while Chen drew the conclusion that low‐grade chondrosarcoma in the spine was resistant to radiotherapy^21^. In our study, we found that radiotherapy was a risk factor for CSS, and further subgroup analysis revealed that patients treated with radiotherapy tended to have greater tumor extensions (*P* < 0.001) and higher grade (*P* < 0.001) (Table S13–S14), concurring with Söderström *et al*.^34^

Based on independent prognostic factors discussed above, we constructed nomograms to predict patients’ survival. To evaluate its generalizability and practical application, we applied the external validation and achieved satisfactory fitting degree (internal validation C‐index: OS, 0.787; CSS, 0.821; external: OS, 0.777; CSS, 0.872). In addition, our nomograms displayed better accuracy than the traditional AJCC sixth (OS, 0.640; CSS, 0.673) and seventh editions (OS, 0.675; CSS, 0.711) for predicting survival probability, so they might be widely used by orthopaedists.

There were several limitations in our study. First, although we included many variables and had a large sample size in our series, there were still some variables with inaccurate information in the SEER database. Second, the SEER database did not include variables such as pathologic fracture, surgical margin status, radiotherapy, and chemotherapy incomplete, which had been known as potential prognostic factors^7^. Third, this is a retrospective study. Fourth, age and gender as prognostic factors were controversial in previous studies. Thus, we had strict inclusion and exclusion criteria to minimize this demographic heterogeneity. We also performed eight subgroup Cox regression analyses (age was divided into two groups: < 65, ≥65) and gender was divided into two groups, showing the significant predictors were stable in different subgroups (Fig. S1‐S8) (chondroblastoma, clear cell chondrosarcoma, and juxtacortical chondrosarcoma were not included in the subgroup analysis because some of these histology subtypes only had one sample in some groups). Finally, owing to the relative limitation of the number of patients in the validation set and the variable heterogeneity between the two sets, the results of external validation might be biased. To explore the sources of the heterogeneity, Table S15 summarizes the baseline characteristics of patients with non‐metastatic chondrosarcoma in training and validation sets, showing demographic homogeneity (Categorical age: *P* = 0.155; Gender: *P* = 0.250). However, statistically significant heterogeneity was found in tumor extension, primary site, grade, ICD‐O‐3 histology subtype, surgery information, chemotherapy, and survival months between the two sets, which might be attributed to the ethnic differences, short follow‐up time and relatively small sample size. In the future, more data should be collected and incorporated to improve the nomogram.

### *Conclusion*

Notwithstanding its limitations, the present study did indicate that our nomograms based on basal clinicopathologic features could well predict the 3‐year and 5‐year survival probability of patients with non‐metastatic chondrosarcoma. In the future, we will endeavor to increase the sample size and extend the follow‐up time. Stricter and more accurate nomograms for prediction need to be contrasted with genetic and epigenetic factors. Subsequent research should focus on incorporating the deep molecular mechanisms and clinical prognosis indicators.

## Supporting information


**Fig. S1** The result of subgroup Cox regression analysis for overall survival (OS) based on the patients less than 65 years old.
**Fig. S2** The result of subgroup Cox regression analysis for cause‐specific survival (CSS) based on the patients less than 65 years old.
**Fig. S3** The result of subgroup Cox regression analysis for overall survival (OS) based on the patients 65 years old or older.
**Fig. S4** The result of subgroup Cox regression analysis for cause‐specific survival (CSS) based on the patients 65 years old or older.
**Fig. S5** The result of subgroup Cox regression analysis for overall survival (OS) based on the male patients.
**Fig. S6** The result of subgroup Cox regression analysis for cause‐specific survival (CSS) based on the male patients.
**Fig. S7** The result of subgroup Cox regression analysis for overall survival (OS) based on the female patients.
**Fig. S8** The result of subgroup Cox regression analysis for cause‐specific survival (CSS) based on the female patients.
**Figure S1‐S8** shows the results of eight subgroup Cox regression analysis (The age was divided into two groups: <65, ≥65) (The gender was divided into two groups).

**Table S1** shows baseline characteristics of patients with non‐metastatic chondrosarcoma.
**Table S2** shows results of single factor analysis and random forest.
**Table S3** shows point assignment and prognostic score for each variable in nomograms.
**Table S4**–**S14** shows the subgroup univariate analysis results.
**Table S15** shows the baseline characteristics of patients with non‐metastatic chondrosarcoma in training and validation set.
