# Supplementary material for: Identifying the Prognosis Factors and Predicting the Survival Probability in Patients with Non‐Metastatic Chondrosarcoma from the SEER Database
Source: Orthop Surg. 2019 Oct 29;11(5):801–10. doi: 10.1111/os.12521 (PMC6819193; doi:10.1111/os.12521)
Supplement: Supplementary file 1 — Fig. S1 The result of subgroup Cox regression analysis for overall survival (OS) based on the patients less than 65 years old. Fig. S2 The result of subgroup Cox regression analysis for cause‐specific survival (CSS) based on the patients less than 65 years old. Fig. S3 The result of subgroup Cox regression analysis for overall survival (OS) based on the patients 65 years old or older. Fig. S4 The result of subgroup Cox regression analysis for cause‐specific survival (CSS) based on the patients 65 years old or older. Fig. S5 The result of subgroup Cox regression analysis for overall survival (OS) based on the male patients. Fig. S6 The result of subgroup Cox regression analysis for cause‐specific survival (CSS) based on the male patients. Fig. S7 The result of subgroup Cox regression analysis for overall survival (OS) based on the female patients. Fig. S8 The result of subgroup Cox regression analysis for cause‐specific survival (CSS) based on the female patients. Figure S1‐S8 shows the results of eight subgroup Cox regression analysis (The age was divided into two groups: <65, ≥65) (The gender was divided into two groups). [file OS-11-801-s001.pdf]

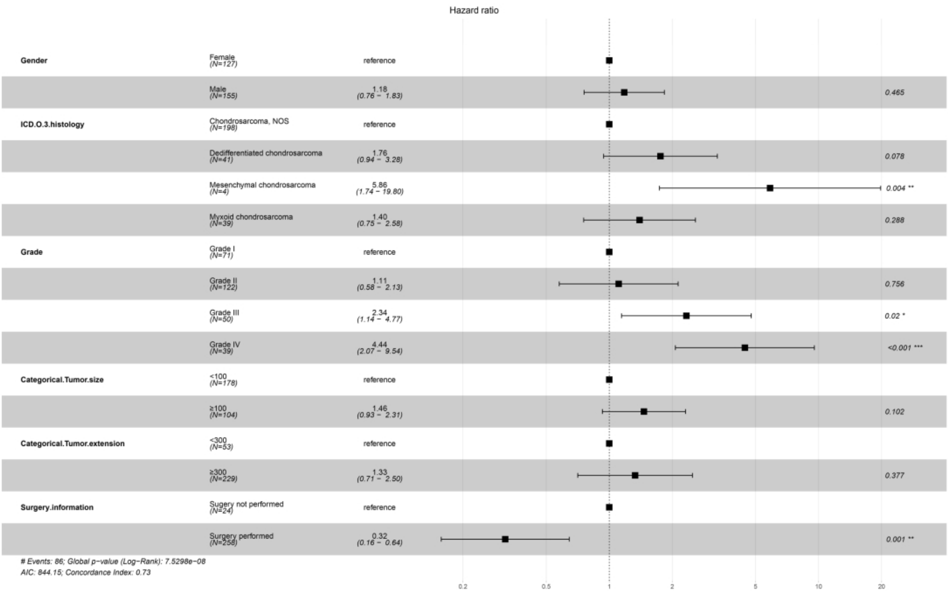

The result of subgroup Cox regression analysis for overall survival (OS) based on the patients less than 65 years old.

299x180mm (300 x 300 DPI)

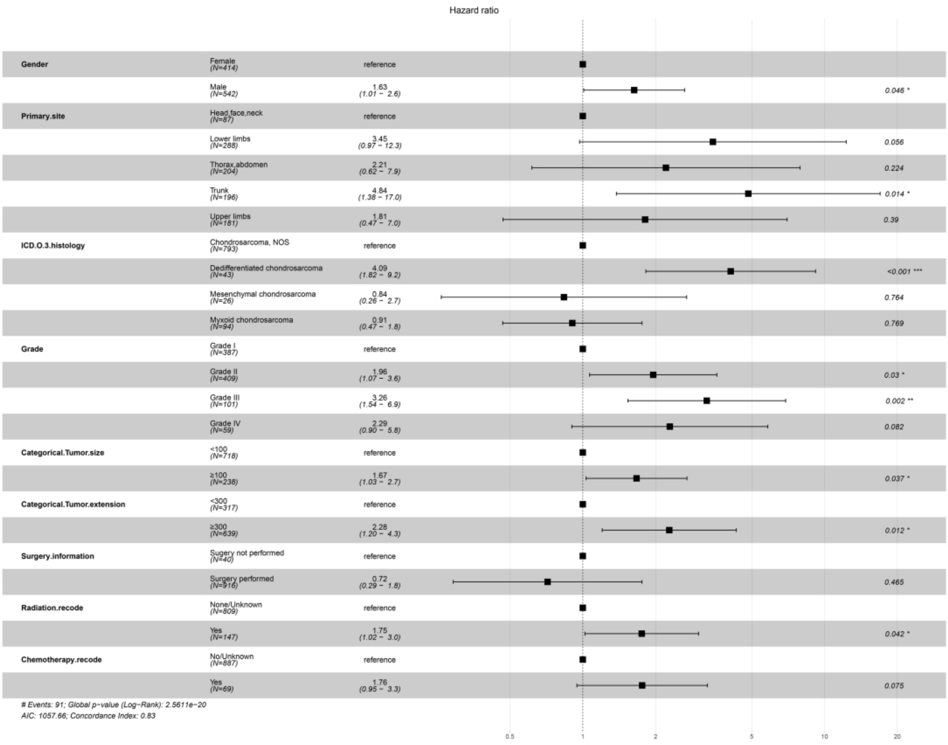

The result of subgroup Cox regression analysis for cause-specific survival (CSS) based on the patients less than 65 years old.

299x224mm (300 x 300 DPI)

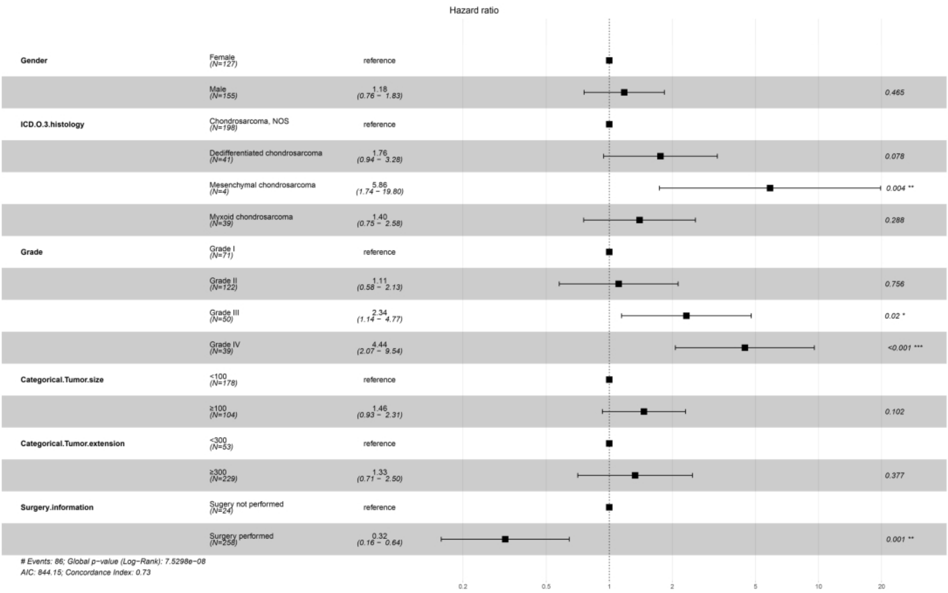

The result of subgroup Cox regression analysis for overall survival (OS) based on the patients 65 years old or older.

299x180mm (300 x 300 DPI)

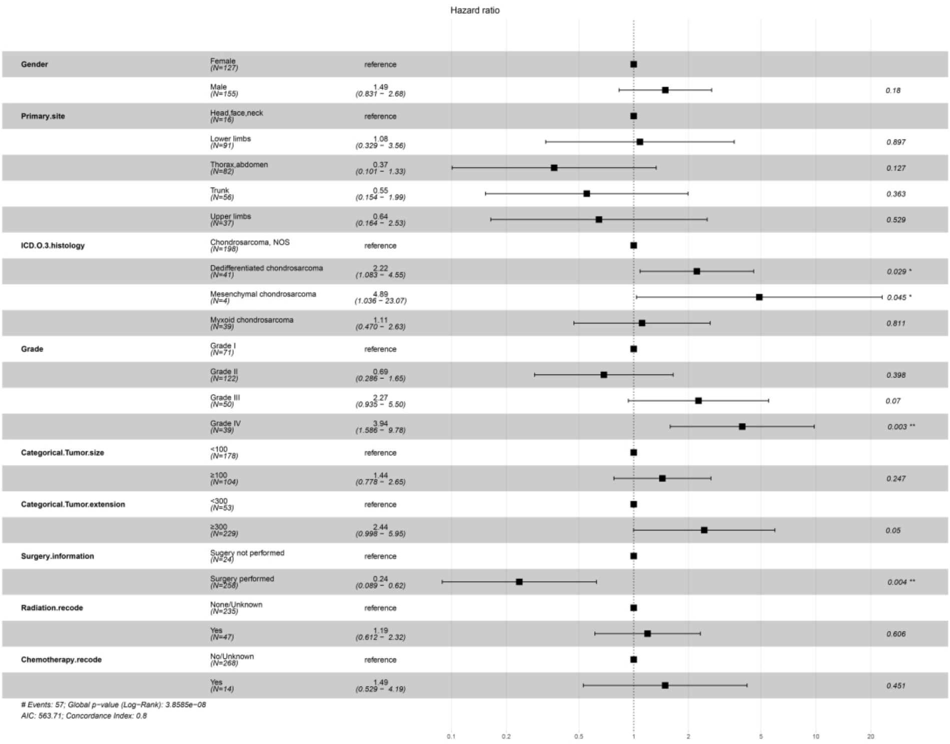

The result of subgroup Cox regression analysis for cause-specific survival (CSS) based on the patients 65 years old or older.

299x224mm (300 x 300 DPI)

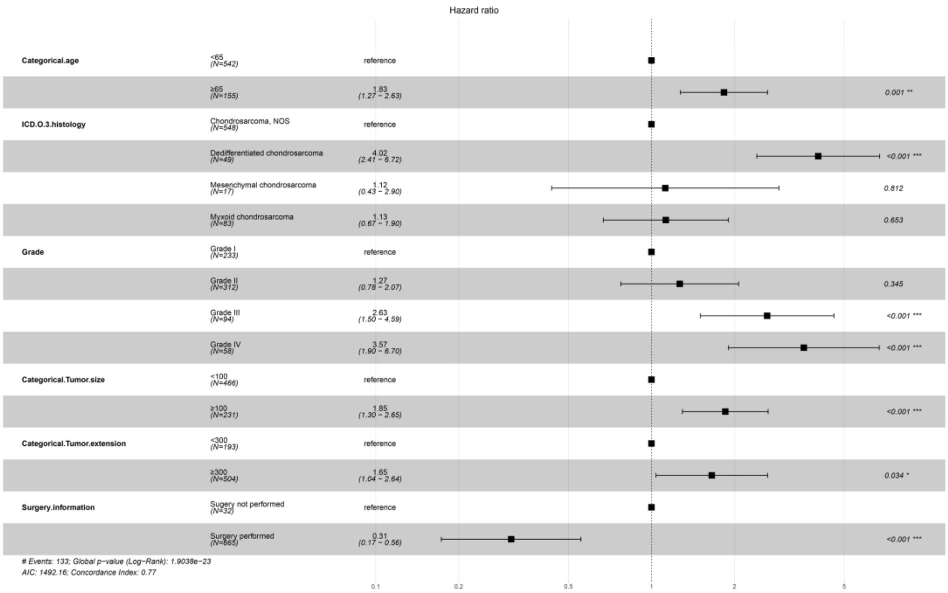

The result of subgroup Cox regression analysis for overall survival (OS) based on the male patients.

299x180mm (300 x 300 DPI)

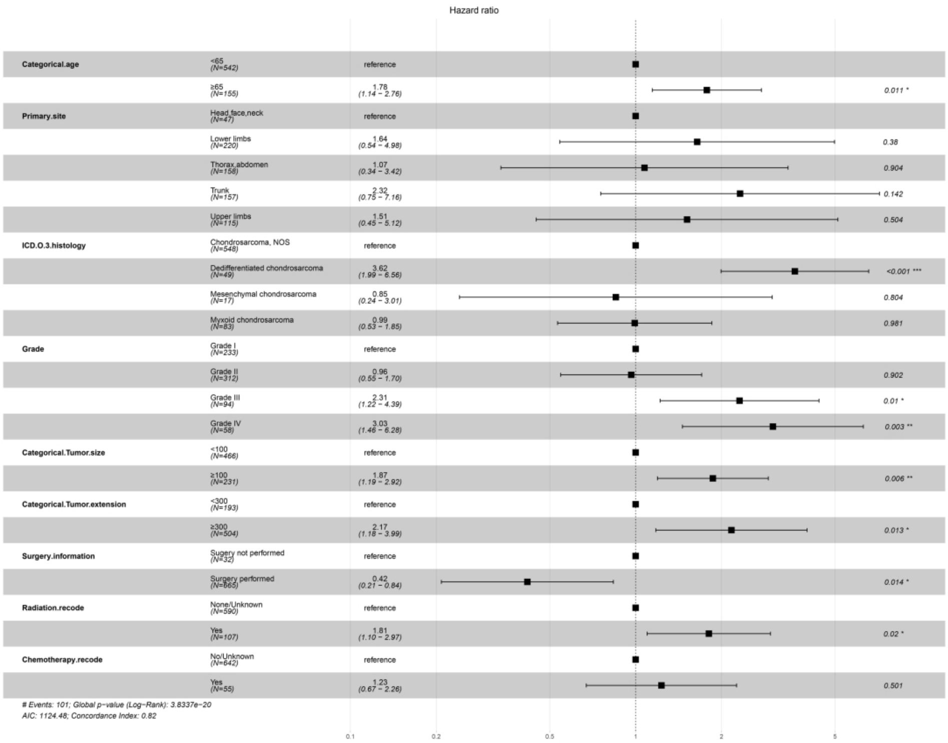

The result of subgroup Cox regression analysis for cause-specific survival (CSS) based on the male patients.

299x224mm (300 x 300 DPI)

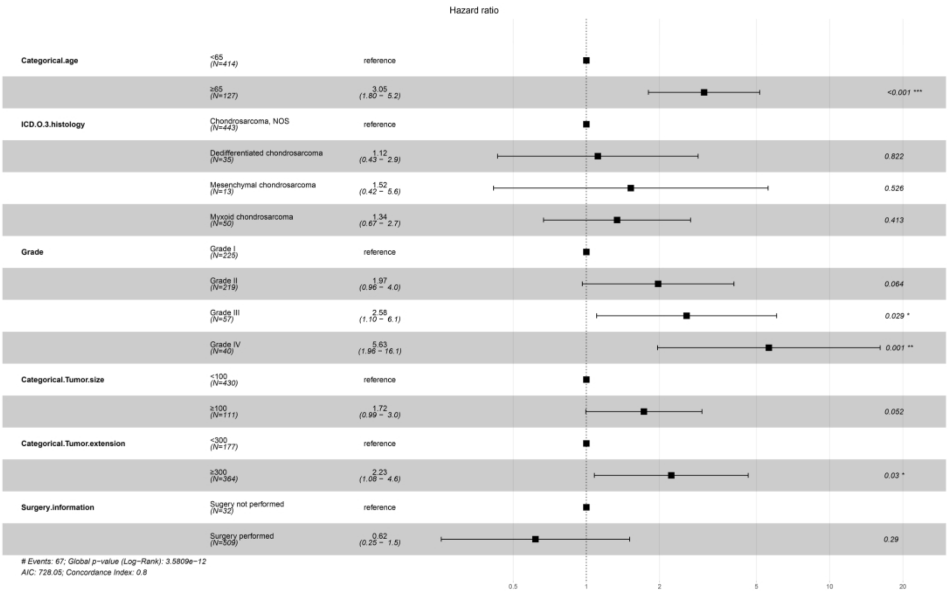

The result of subgroup Cox regression analysis for overall survival (OS) based on the female patients.

299x180mm (300 x 300 DPI)

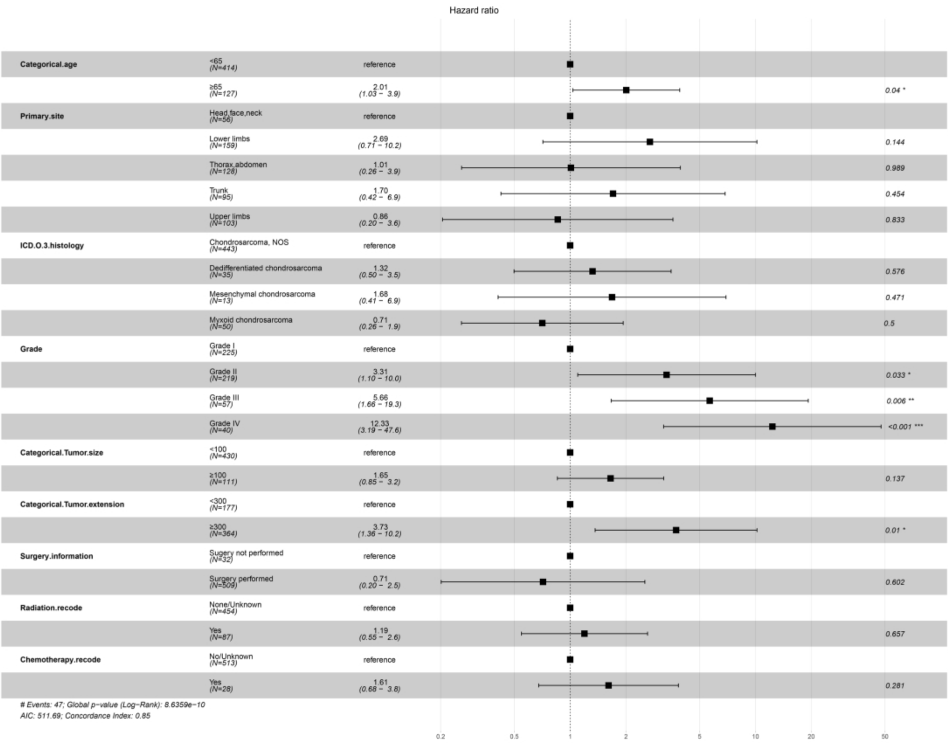

The result of subgroup Cox regression analysis for cause-specific survival (CSS) based on the female patients.

299x224mm (300 x 300 DPI)
