# Supplementary material for: Identifying the Prognosis Factors and Predicting the Survival Probability in Patients with Non‐Metastatic Chondrosarcoma from the SEER Database
Source: Orthop Surg. 2019 Oct 29;11(5):801–10. doi: 10.1111/os.12521 (PMC6819193; doi:10.1111/os.12521)
Supplement: Supplementary file 2 — Table S1 shows baseline characteristics of patients with non‐metastatic chondrosarcoma. Table S2 shows results of single factor analysis and random forest. Table S3 shows point assignment and prognostic score for each variable in nomograms. Table S4–S14 shows the subgroup univariate analysis results. Table S15 shows the baseline characteristics of patients with non‐metastatic chondrosarcoma in training and validation set. [file OS-11-801-s002.docx]

**Appendix:**

Supplementary materials A

Table S1 shows baseline characteristics of patients with non-metastatic chondrosarcoma.

Table S2 shows results of single factor analysis and random forest

Table S3 shows point assignment and prognostic score for each variable in nomograms.

Table S4 – S14 shows the subgroup univariate analysis results.

Table S15 shows the baseline characteristics of patients with non-metastatic chondrosarcoma in training and validation set.

Figure S1-S8 shows the results of eight subgroup Cox regression analysis (The age was divided into two groups: < 65, ≥ 65) (The gender was divided into two groups).

Supplementary materials B

The raw dataset of the training set consisted of 1267 patients with non-metastatic chondrosarcoma from SEER database

Supplementary materials C

The raw dataset of the validated set consisted of 72 patients with non-metastatic chondrosarcoma from the First Affiliated Hospital of Zhengzhou University.

**Table S1** Baseline characteristics of patients with non-metastatic chondrosarcoma.

| Demographic or Characteristic | Total Patients (N=1267) | | Alive cohort(N=1065) | | Dead cohort(N=202) | |
| --- | --- | --- | --- | --- | --- | --- |
|  | No. | % | No. | % | No. | % |
| Age, years |  |  |  |  |  |  |
| Mean ± SD | 50.51 ± 17.04 | | 48.76 ± 16.68 | | 59.71 ± 15.94 | |
| Median (Range) | 51.00(4.00-91.00) | | 49.00(4.00-88.00) | | 61.00(10.00-91.00) | |
| Categorical Age |  |  |  |  |  |  |
| <65 | 983 | 77.6% | 867 | 81.4% | 116 | 57.4% |
| ≥65 | 284 | 22.4% | 198 | 18.6% | 86 | 42.6% |
| Gender |  |  |  |  |  |  |
| female | 546 | 43.1% | 479 | 45.0% | 67 | 33.2% |
| male | 721 | 56.9% | 586 | 55.0% | 135 | 66.8% |
| Race |  |  |  |  |  |  |
| Black | 93 | 7.3% | 82 | 7.7% | 11 | 5.4% |
| Other | 70 | 5.5% | 64 | 6.0% | 6 | 3.0% |
| White | 1104 | 87.1% | 919 | 86.3% | 185 | 91.6% |
| Tumor size, mm |  |  |  |  |  |  |
| Mean ± SD | 78.77 ± 58.45 | | 73.08 ± 54.11 | | 108.79 ± 70.16 | |
| median (Range) | 65.00(4.00-890.00) | | 60.00(4.00-890.00) | | 95.00(7.00-430.00) | |
| Categorical tumor size |  |  |  |  |  |  |
| <100mm | 918 | 72.5% | 813 | 76.3% | 105 | 52.0% |
| ≥100mm | 349 | 27.5% | 252 | 23.7% | 97 | 48.0% |
| Tumor extension |  |  |  |  |  |  |
| Mean ± SD | 328.16 ± 174.56 | | 312.98 ± 169.33 | | 408.22 ± 179.78 | |
| Median(Range) | 300.00(100.00-850.00) | | 300.00(100.00-850.00) | | 400.00(100.00-820.00) | |
| Categorical tumor extension |  |  |  |  |  |  |
| <300 | 379 | 29.9% | 346 | 32.5% | 33 | 16.3% |
| ≥300 | 888 | 70.1% | 719 | 67.5% | 169 | 83.7% |
| Primary Site |  |  |  |  |  |  |
| Head, face, neck | 103 | 8.1% | 93 | 8.7% | 10 | 5.0% |
| Lower limb | 398 | 31.4% | 324 | 30.4% | 74 | 36.6% |
| Thorax, abdomen | 287 | 22.7% | 252 | 23.7% | 35 | 17.3% |
| Trunk | 257 | 20.3% | 201 | 18.9% | 56 | 27.7% |
| Upper limb | 222 | 17.5% | 195 | 18.3% | 27 | 13.4% |
| Grade |  |  |  |  |  |  |
| Grade I | 469 | 37.0% | 432 | 40.6% | 37 | 18.3% |
| Grade II | 542 | 42.8% | 463 | 43.5% | 79 | 39.1% |
| Grade III | 157 | 12.4% | 111 | 10.4% | 46 | 22.8% |
| Grade IV | 99 | 7.8% | 59 | 5.5% | 40 | 19.8% |
| ICD-O-3 histology |  |  |  |  |  |  |
| Chondrosarcoma, NOS | 991 | 78.2% | 868 | 81.5% | 123 | 60.9% |
| Dedifferentiated chondrosarcoma | 84 | 6.6% | 44 | 4.1% | 40 | 19.8% |
| Mesenchymal chondrosarcoma | 30 | 2.4% | 22 | 2.1% | 8 | 4.0% |
| Myxoid chondrosarcoma | 133 | 10.5% | 104 | 9.8% | 29 | 14.4% |
| Others | 29 | 2.3% | 27 | 2.5% | 2 | 1.0% |
| AJCC T |  |  |  |  |  |  |
| T1 | 772 | 60.9% | 693 | 65.1% | 79 | 39.1% |
| T2 | 484 | 38.2% | 364 | 34.2% | 120 | 59.4% |
| T3 | 11 | .9% | 8 | .8% | 3 | 1.5% |
| Surgery information |  |  |  |  |  |  |
| Surgery not performed | 64 | 5.1% | 43 | 4.0% | 21 | 10.4% |
| Surgery performed | 1203 | 94.9% | 1022 | 96.0% | 181 | 89.6% |
| Radiation |  |  |  |  |  |  |
| No/Unknown | 1073 | 84.7% | 916 | 86.0% | 157 | 77.7% |
| Yes | 194 | 15.3% | 149 | 14.0% | 45 | 22.3% |
| Chemotherapy |  |  |  |  |  |  |
| No/Unknown | 1183 | 93.4% | 1012 | 95.0% | 171 | 84.7% |
| Yes | 84 | 6.60% | 53 | 5.0% | 31 | 15.3% |
| Survival months |  |  |  |  |  |  |
| Mean ± SD | 48.63±34.49 | | 52.29±35.08 | | 29.34±23.04 | |
| Median (Range) | 42.00(0.00-119.00) | | 49.00(0.00-119.00) | | 23.00(0.00-109.00) | |
| Marital status |  |  |  |  |  |  |
| Married | 772 | 60.9% | 657 | 61.7% | 115 | 56.9% |
| Single/Separated/Divorced/Widowed | 495 | 39.1% | 408 | 38.3% | 87 | 43.1% |
| 9th grade education | |  |  |  |  |  |
| Lower 50% | 629 | 49.6% | 520 | 48.8% | 109 | 54.0% |
| Upper 50% | 638 | 50.4% | 545 | 51.2% | 93 | 46.0% |
| High school education | |  |  |  |  |  |
| Lower 50% | 328 | 25.9% | 282 | 26.5% | 46 | 22.8% |
| Upper 50% | 939 | 74.1% | 783 | 73.5% | 156 | 77.2% |
| At least bachelors degree | |  |  |  |  |  |
| Lower 50% | 484 | 38.2% | 407 | 38.2% | 77 | 38.1% |
| Upper 50% | 783 | 61.8% | 658 | 61.8% | 125 | 61.9% |
| Median family income | |  |  |  |  |  |
| Lower 50% | 628 | 49.6% | 514 | 48.3% | 114 | 56.4% |
| Upper 50% | 639 | 50.4% | 551 | 51.7% | 88 | 43.6% |
| Families below poverty | |  |  |  |  |  |
| Lower 50% | 632 | 49.9% | 540 | 50.7% | 92 | 45.5% |
| Upper 50% | 635 | 50.1% | 525 | 49.3% | 110 | 54.5% |
| Unemployed |  |  |  |  |  |  |
| Lower 50% | 631 | 49.8% | 544 | 51.1% | 87 | 43.1% |
| Upper 50% | 636 | 50.2% | 521 | 48.9% | 115 | 56.9% |
| White collar |  |  |  |  |  |  |
| Lower 50% | 542 | 42.8% | 455 | 42.7% | 87 | 43.1% |
| Upper 50% | 725 | 57.2% | 610 | 57.3% | 115 | 56.9% |

**Abbreviations:** OS, overall survival; CSS, cause-specific survival; AJCC, American Joint Committee on Cancer; NOS, no other specific;

**Table S2** Results of single factor analysis and random forest

| Variables | Overall survival (OS) | | | | Cancer specific survival (CSS) | | | |
| --- | --- | --- | --- | --- | --- | --- | --- | --- |
|  | P value of parameter or non-parametric test | P value of Kaplan-meier survival analysis | MDG |  | P value of parameter or non-parametric test | P value of Kaplan-meier survival analysis | MDG |  |
| Age | < 0.001* | < 0.001* | 15.897613 | | < 0.001* | < 0.001* | 9.416718 | |
| Tumor size | < 0.001* | < 0.001* | 10.753939 | | < 0.001* | < 0.001* | 8.485833 | |
| Tumor extension | < 0.001* | < 0.001* | 10.124286 | | < 0.001* | < 0.001* | 7.524938 | |
| Race | 0.102 | 0.140 | 8.684427 | | 0.357 | 0.390 | 6.696191 | |
| Gender | 0.002* | 0.002* | 12.968371 | | 0.002* | 0.002* | 9.405549 | |
| Primary site | 0.002* | 0.001* | 31.17838 | | < 0.001* | < 0.001* | 23.437642 | |
| Grade | < 0.001* | < 0.001* | 32.189324 | | < 0.001* | < 0.001* | 27.553934 | |
| ICD.O.3.Histology | < 0.001* | < 0.001* | 28.754762 | | < 0.001* | < 0.001* | 22.927376 | |
| AJCC.T | < 0.001* | < 0.001* | 11.832568 | | < 0.001* | < 0.001* | 8.890352 | |
| Surgery information | < 0.001* | < 0.001* | 7.778551 | | 0.010* | 0.002* | 4.880497 | |
| Radiation | 0.003* | 0.002* | 9.192384 | | 0.001* | < 0.001* | 8.308236 | |
| Chemotherapy | < 0.001* | < 0.001* | 9.50855 | | < 0.001* | < 0.001* | 10.115413 | |
| Marital status | 0.204 | 0.069 | 13.048278 | | 0.556 | 0.970 | 9.551751 | |
| X9th grade education | 0.181 | 0.370 | 12.252002 | | 0.293 | 0.480 | 9.228653 | |
| High school education | 0.270 | 0.140 | 8.193728 | | 0.477 | 0.300 | 6.239044 | |
| At least bachelors degree | 0.979 | 0.960 | 9.29573 | | 0.275 | 0.290 | 6.987954 | |
| Media family income | 0.330 | 0.014* | 10.139049 | | 0.052 | 0.026* | 7.982188 | |
| Families below poverty | 0.179 | 0.067 | 8.785488 | | 0.353 | 0.180 | 6.644799 | |
| Unemployed | 0.037* | 0.021* | 10.441735 | | 0.051 | 0.029* | 8.23682 | |
| White collar | 0.037* | 0.860 | 9.370591 | | 0.453 | 0.450 | 6.893658 | |

**NOTE:** Categorical variables were compared by using the Pearson Chi-square test. Continuous variables in normal distribution and homogeneity of variance were compared by using the two-sample t test, otherwise, the Mann-Whitney U test were performed. Out of bag (OOB) error rate for OS and CSS was 16.73% and 12.63%, respectively.

**Abbreviations:** OS, overall survival; CSS, cause-specific survival; MDG, Mean Decrease Gini; AJCC, American Joint Committee on Cancer.

* P < 0.05.

**Table S3** Point assignment and prognostic score for each variable

| Variable | OS | CCS |
| --- | --- | --- |
| Categorical age |  |  |
| < 65 | 0 | 0 |
| ≥ 65 | 42 | 28 |
| Categorical tumor size |  |  |
| < 100 | 0 | 0 |
| ≥ 100 | 33 | 24 |
| Categorical tumor extension |  |  |
| < 300 | 0 | 0 |
| ≥ 300 | 30 | 39 |
| Sex |  |  |
| Female | 0 | 0 |
| Male | 27 | 20 |
| Primary site |  |  |
| Head, face, neck |  | 1 |
| Lower limb |  | 30 |
| Thorax, abdomen |  | 0 |
| Trunk |  | 34 |
| Upper limb |  | 4 |
| Grade |  |  |
| Grade I | 0 | 0 |
| Grade II | 22 | 14 |
| Grade III | 57 | 47 |
| Grade IV | 76 | 62 |
| Histological subtype |  |  |
| Chondrosarcoma, NOS | 43 | 58 |
| Dedifferentiated | 100 | 100 |
| Mesenchymal | 57 | 59 |
| Myxoid | 54 | 53 |
| Others | 0 | 0 |
| Surgery information |  |  |
| Yes | 0 | 0 |
| No | 37 | 52 |
| Chemotherapy |  |  |
| Yes |  | 18 |
| No/Unknown |  | 0 |
| Radiotherapy |  |  |
| Yes |  | 18 |
| No/Unknown |  | 0 |

**Abbreviations:** OS, overall survival; CSS, cause-specific survival; ICD, International Classification of Diseases;

**Table S4** Results of univariate analysis between surgery information and categorical age

| Surgery information | Categorical Age | OR | 95%CI | P value |
| --- | --- | --- | --- | --- |
| No | < 65 | 1.000 (reference) |  |  |
|  | ≥ 65 | 3.443 | 1.549 - 9.159 | 0.005* |

**NOTE:** Performing the surgery is the reference group.

**Abbreviation:** OR, odds ratio; CI, confidence interval;

*P < 0.05

**Table S5** Results of univariate analysis between tumor size and gender

| Tumor size (mm) | Gender | OR | 95%CI | P value |
| --- | --- | --- | --- | --- |
| ≥ 100 | Female | 1.000 (reference) |  |  |
|  | Male | 1.931 | 1.492 - 2.510 | < 0.001* |

**NOTE**: Tumor size < 100 is the reference group.

**Abbreviation:** OR, odds ratio; CI, confidence interval;

*P < 0.05

**Table S6** Results of univariate analysis between primary site and gender

| Primary site | Gender | OR | 95%CI | P value |
| --- | --- | --- | --- | --- |
| Lower limb | Female | 1.000 (reference) |  |  |
|  | Male | 1.718 | 1.111 - 2.657 | 0.015* |
| Thorax, abdomen | Female | 1.000 (reference) |  |  |
|  | Male | 1.480 | 0.942 - 2.326 | 0.089 |
| Trunk | Female | 1.000 (reference) |  |  |
|  | Male | 1.998 | 1.258 - 3.174 | 0.003* |
| Upper limb | Female | 1.000 (reference) |  |  |
|  | Male | 1.377 | 0.861 - 2.200 | 0.182* |

**NOTE:** Head, face, neck is the reference group.

**Abbreviation:** OR, odds ratio; CI, confidence interval;

*P < 0.05

**Table S7** Results of univariate analysis between surgery information and tumor extension

| Surgery information | tumor extension | OR | 95%CI | P value |
| --- | --- | --- | --- | --- |
| Yes | < 300 | 1.000 (reference) |  |  |
|  | ≥ 300 | 0.627 | 0.394 - 0.981 | 0.045* |

**NOTE:** No surgery is the reference group.

**Abbreviation:** OR, odds ratio; CI, confidence interval;

*P < 0.05

**Table S8** Results of univariate analysis between grade and tumor extension

| Grade | tumor extension | OR | 95%CI | P value |
| --- | --- | --- | --- | --- |
| High grade | < 300 | 1.000 (reference) |  |  |
|  | ≥ 300 | 1.724 | 1.252 - 2.406 | 0.001* |

**NOTE:** Low grade is the reference group.

**Abbreviation:** OR, odds ratio; CI, confidence interval;

*P < 0.05

**Table S9** Results of univariate analysis between surgery information and primary site

| Surgery information | Primary site | OR | 95%CI | P value |
| --- | --- | --- | --- | --- |
| No | Head, face, neck | 1.000 (reference) |  |  |
|  | Lower limb | 1.579 | 1.549 - 9.159 | 0.357 |
|  | Thorax, abdomen | 2.157 | 0.597 - 4.177 | 0.164 |
|  | Trunk | 0.601 | 0.730 - 6.374 | 0.280 |
|  | Upper limb | 1.187 | 0.426 - 3.301 | 0.743 |

**NOTE:** Performing the surgery is the reference group.

**Abbreviation:** OR, odds ratio; CI, confidence interval;

*P < 0.05

**Table S10** Results of univariate analysis between grade and chemotherapy

| Grade | Chemotherapy | OR | 95%CI | P value |
| --- | --- | --- | --- | --- |
| Lower grade | None/Unknown | 1.000 (reference) |  |  |
|  | Yes | 0.108 | 0.066 - 0.172 | < 0.001* |

**NOTE:** High grade is the reference group.

**Abbreviation:** OR, odds ratio; CI, confidence interval;

*P < 0.05

**Table S11** Results of univariate analysis between tumor size and chemotherapy

| Tumor size (mm) | Chemotherapy | OR | 95%CI | P value |
| --- | --- | --- | --- | --- |
| ≥ 100 | No/Unknown | 1.000 (reference) |  |  |
|  | Yes | 4.590 | 2.920 - 7.310 | < 0.001* |

**NOTE:** Tumor size<100 is the reference group.

**Abbreviation:** OR, odds ratio; CI, confidence interval;

*P < 0.05

**Table S12** Results of univariate analysis between tumor extension and chemotherapy

| Tumor extension (mm) | Chemotherapy | OR | 95%CI | P value |
| --- | --- | --- | --- | --- |
| ≥ 300 | No/Unknown | 1.000 (reference) |  |  |
|  | Yes | 2.669 | 1.502 - 5.285 | 0.002* |

**NOTE:** Tumor extension < 300 is the reference group.

**Abbreviation:** OR, odds ratio; CI, confidence interval;

*P < 0.05

**Table S13** Results of univariate analysis between tumor extension and radiotherapy

| Tumor extension (mm) | Radiotherapy | OR | 95%CI | P value |
| --- | --- | --- | --- | --- |
| ≥300 | No/Unknown | 1.000 (reference) |  |  |
|  | Yes | 1.985 | 1.371 - 2.941 | < 0.001* |

**NOTE:** Tumor extension < 300 is the reference group.

**Abbreviation:** OR, odds ratio; CI, confidence interval;

*P < 0.05

**Table S14** Results of univariate analysis between grade and radiotherapy

| Grade | Radiotherapy | OR | 95%CI | P value |
| --- | --- | --- | --- | --- |
| Lower grade | No/Unknown | 1.000 (reference) |  |  |
|  | Yes | 0.331 | 0.238 - 0.462 | < 0.001* |

**NOTE:** High grade is the reference group.

**Abbreviation:** OR, odds ratio; CI, confidence interval;

*P < 0.05

**Table S15** Baseline characteristics of patients with non-metastatic chondrosarcoma in training and validation set

| Demographic or Characteristic | Training set (N=1267) | | Validation set (N=42) | | P value |
| --- | --- | --- | --- | --- | --- |
|  | No. | % | No. | % |  |
| Categorical Age |  |  |  |  | 0.155 |
| <65 | 983 | 77.6% | 61 | 84.7% |  |
| ≥65 | 284 | 22.4% | 11 | 15.3% |  |
| Gender |  |  |  |  | 0.250 |
| Female | 546 | 43.1% | 36 | 50.0% |  |
| Male | 721 | 56.9% | 36 | 50.0% |  |
| Tumor size |  |  |  |  | 0.966 |
| <100 mm | 918 | 72.5% | 52 | 72.2% |  |
| ≥100mm | 349 | 27.5% | 20 | 27.8% |  |
| Tumor extension |  |  |  |  | < 0.001 |
| <300 mm | 379 | 29.9% | 70 | 97.2% |  |
| ≥300 mm | 888 | 70.1% | 2 | 2.8% |  |
| Primary Site |  |  |  |  | < 0.001 |
| Head, face, neck | 103 | 8.1% | 21 | 29.2% |  |
| Lower limb | 398 | 31.4% | 11 | 15.3% |  |
| Thorax, abdomen | 287 | 22.7% | 17 | 23.6% |  |
| Trunk | 257 | 20.3% | 19 | 26.4% |  |
| Upper limb | 222 | 17.5% | 4 | 5.6% |  |
| Grade |  |  |  |  | < 0.001 |
| Grade I | 469 | 37.0% | 43 | 59.7% |  |
| Grade II | 542 | 42.8% | 13 | 18.1% |  |
| Grade III | 157 | 12.4% | 8 | 11.1% |  |
| Grade IV | 99 | 7.8% | 8 | 11.1% |  |
| ICD-O-3 histology |  |  |  |  | < 0.001 |
| Chondrosarcoma, NOS | 991 | 78.2% | 58 | 80.6% |  |
| Dedifferentiated chondrosarcoma | 84 | 6.6% | 1 | 1.4% |  |
| Mesenchymal chondrosarcoma | 30 | 2.4% | 7 | 9.7% |  |
| Myxoid chondrosarcoma | 133 | 10.5% | 2 | 2.8% |  |
| Others | 29 | 2.3% | 4 | 5.6% |  |
| Surgery information |  |  |  |  | < 0.001 |
| Surgery not performed | 64 | 5.1% | 5 | 6.9% |  |
| Surgery performed | 1203 | 94.9% | 67 | 93.1% |  |
| Radiation |  |  |  |  | 0.531 |
| No/Unknown | 1073 | 84.7% | 59 | 81.9% |  |
| Yes | 194 | 15.3% | 13 | 18.1% |  |
| Chemotherapy |  |  |  |  | < 0.001 |
| No/Unknown | 1183 | 93.4% | 59 | 81.9% |  |
| Yes | 84 | 6.60% | 13 | 18.1% |  |
| Survival months |  |  |  |  | < 0.001 |
| Mean ± SD | 48.63±34.49 | | 29.17±24.46 | |  |
| Median (Range) | 42.00(0.00-119.00) | | 20.50(1.00-92.00) | |  |

**Abbreviations:** OS, overall survival; CSS, cause-specific survival; AJCC, American Joint Committee on Cancer; NOS, no other specific.
